# Detection, Classification, and Segmentation of Rib Fractures From CT Data Using Deep Learning Models: A Review of Literature and Pooled Analysis

**DOI:** 10.1097/RTI.0000000000000833

**Published:** 2025-05-23

**Authors:** Stella Den Hengst, Noor Borren, Esther M.M. Van Lieshout, Job N. Doornberg, Theo Van Walsum, Mathieu M.E. Wijffels, Michael H.J. Verhofstad

**Affiliations:** * Trauma Research Unit, Department of Surgery, Erasmus MC, University Medical Center; ‡ Department of Radiology & Nuclear Medicine, Biomedical Imaging Group Rotterdam, Erasmus MC, Rotterdam; † Department of Trauma Surgery, University Medical Centre Groningen and Groningen University, Groningen, The Netherlands

**Keywords:** artificial intelligence, deep learning, rib fracture, computed tomography

## Abstract

**Purpose:**

Trauma-induced rib fractures are common injuries. The gold standard for diagnosing rib fractures is computed tomography (CT), but the sensitivity in the acute setting is low, and interpreting CT slices is labor-intensive. This has led to the development of new diagnostic approaches leveraging deep learning (DL) models. This systematic review and pooled analysis aimed to compare the performance of DL models in the detection, segmentation, and classification of rib fractures based on CT scans.

**Materials and Methods:**

A literature search was performed using various databases for studies describing DL models detecting, segmenting, or classifying rib fractures from CT data. Reported performance metrics included sensitivity, false-positive rate, F1-score, precision, accuracy, and mean average precision. A meta-analysis was performed on the sensitivity scores to compare the DL models with clinicians.

**Results:**

Of the 323 identified records, 25 were included. Twenty-one studies reported on detection, four on segmentation, and 10 on classification. Twenty studies had adequate data for meta-analysis. The gold standard labels were provided by clinicians who were radiologists and orthopedic surgeons. For detecting rib fractures, DL models had a higher sensitivity (86.7%; 95% CI: 82.6%-90.2%) than clinicians (75.4%; 95% CI: 68.1%-82.1%). In classification, the sensitivity of DL models for displaced rib fractures (97.3%; 95% CI: 95.6%-98.5%) was significantly better than that of clinicians (88.2%; 95% CI: 84.8%-91.3%).

**Conclusions:**

DL models for rib fracture detection and classification achieved promising results. With better sensitivities than clinicians for detecting and classifying displaced rib fractures, the future should focus on implementing DL models in daily clinics.

**Level of Evidence:**

Level III—systematic review and pooled analysis

Trauma-induced rib fractures are common injuries that affect millions of individuals globally each year, with a prevalence of 10% to 40% in all trauma patients.^1–4^ Common causes in all trauma patients are high-energy trauma (HET) and low-energy trauma (LET) in older patients.^5^ In general, rib fractures have high morbidity, and the combination with other conditions, such as hemothorax, pneumothorax, extremity fractures, and injuries to soft tissue, increases the mortality risk.^1^ Moreover, inadequate pain control can lead to respiratory complications, such as pneumonia, due to impaired coughing and insufficient breathing.^6^ Treatment strategies should be tailored to both rib fracture characteristics, such as the type and number of rib fractures, and patient characteristics, such as age and comorbidities.^7,8^

In the primary survey of trauma patients, anteroposterior thoracic radiography is the standard of care. However, the sensitivity of a thoracic radiograph is low, and 50% to 80% of rib fractures remain undetected.^9–11^ Therefore, the gold standard for diagnosing rib fractures is a computed tomography (CT) scan. Although this improves the sensitivity, 19.2% to 26.8% of the rib fractures are still missed.^12–14^ In addition to the clinical importance, CT scans are vital for rib fracture classification systems. Ideally, classification systems predict clinical outcomes, enable clear and uniform communication, and support scientific research.^15^ Unfortunately, current literature shows large interobserver variability (*κ* = 0.46 [95% CI: 0.32-0.59]), if classification is performed by clinicians, suggesting the need for better CT-scoring entities.^16^

Low sensitivity and significant interobserver variability exist throughout the medical imaging field. Artificial Intelligence (AI) has shown great potential for aiding clinicians by improving time efficiency, reproducibility, and accuracy.^17^ Machine learning (ML) is a subset of AI. Traditional ML models are dependent on developers who need to quantify features that are used as inputs for a classifier. The prediction of the model is then made on the adjusted weights of these features. In deep learning (DL), however, the model performs the feature extraction itself.^18^ As a result, DL models often show superior performance, particularly in imaging tasks.^17,18^

Three types of DL models are described: detection, segmentation, and classification. A detection model often uses a bounding box or only notes the presence of a rib fracture, without further spatial information. Segmentation models provide more spatial information by delineating the contour of rib fractures. In this review, a model was marked as a segmentation model when the segmentation was centered around the fracture, instead of segmenting the entire ribcage. Classification models classify rib fractures into various classes or based on the presence of fractures.

In the last 3 years, the use of DL models for rib fracture detection has increased. The aim of this literature review and pooled analysis was to answer the following questions: (1) What is the accuracy of DL models in rib fracture detection, segmentation, and classification using CT data? (2) Are DL models superior to clinicians in rib fracture detection, segmentation, and classification using CT data?

## MATERIALS AND METHODS

### Study Selection

Preferred Reporting Items for Systematic Reviews and Meta-Analyses (PRISMA) guidelines were followed in this review.^19^ A systematic literature search was performed on March 27, 2023, using the following databases: Medline ALL, Embase, Web of Science Core Collection, Cochrane Central Register of Controlled Trials databases, and an additional search in Google Scholar. The search strategy was formulated and performed using a librarian (supplemental digital content (SDC) 1, http://links.lww.com/JTI/A334). This protocol was developed before this study.

The inclusion criteria were publications that contain: (1) rib fractures, (2) CT data, and (3) description of a DL model for imaging. The exclusion criteria were publications that were commentaries, conference abstracts, studies not conducted on living humans, nonoriginal studies, and studies not published in English or Dutch. After removing duplicates, titles and abstracts of potentially eligible records were independently screened by 2 reviewers (N.B. and S.D.H.). Subsequently, full-text screening was performed using the inclusion criteria to check for eligibility. A third reviewer (M.M.E.W.) was consulted in cases in which the 2 reviewers did not reach an agreement until a consensus was reached.

### Quality Assessment

Two reviewers (N.B. and S.D.H.) independently assessed the quality of all included articles according to a modified version of the Methodological Index for Non-Randomized Studies (MINORS).^20^ The modified version of MINORS includes the following items: study aim, data selection/input features, (type of) AI model, external test data set, gold standard, demographic report, data set distribution, and adequate statistical analysis/performance metric. The items were scored 0 (not reported) or 1 (reported), with a maximum score of 8. In case of disagreement, a consensus meeting was organized, in which inconsistencies were resolved under the supervision of a third reviewer (M.M.E.W.).

### Data Extraction

Two reviewers (N.B. and S.D.H.) independently extracted the data from the included studies. The extracted data for each study were year of publication, number of patients and fractures, type of AI model used, train-validation-test split of the data set, presence of testing on an external data set (yes/no/not reported), reports about demographics (yes/no/not reported), data selection performed (yes/no/not reported), number of different CT scanners used, slice thickness (in mm), and gold standard label assignment.

### Outcome Measures

The primary outcomes for the detection DL model were performance metrics such as sensitivity, precision, false positives (FP) per patient with the rib fracture as a 3D object or per slice with the rib fracture as a 2D image, F1-score, accuracy, and mean average precision (mAP). The mean sensitivity with SD was calculated. The outcome measures for the segmentation models were sensitivity, specificity, Dice Similarity Coefficient (DSC), and Intersection-over-Union (IoU). The classification models were evaluated for sensitivity, precision, F1-score, and accuracy. All outcome measures were taken from the test set whenever possible. If the sensitivity, precision, or specificity was not given, confusion matrices were noted, and the mentioned values were calculated (SDC 2, Supplemental Digital Content 1, http://links.lww.com/JTI/A334).

To benchmark the accuracy of the DL models, the outcome parameters obtained by the clinicians were extracted from the studies.

### Statistical Analysis

A pooled analysis of the sensitivity scores to compare the DL outcome parameters with those achieved by clinicians was performed using MedCalc.^21^ The internal test set was used for the different DL models, and an additional pooled analysis was done on the external data sets for the DL detection models. Heterogeneity, Cochran Q, and *I*^2^ values were calculated. A random-effects model was used to calculate the proportion because of the expected heterogeneity across the studies. The CI of the DL models and clinicians were compared. The differences were considered statistically significant if the CIs did not overlap. There was no significant difference if the CIs exceeded the middle of the other CI. If the CIs overlapped but did not pass the middle, the difference between the DL models and clinicians remained undecided.^22^

## RESULTS

### Included Studies

**FIGURE 1:**
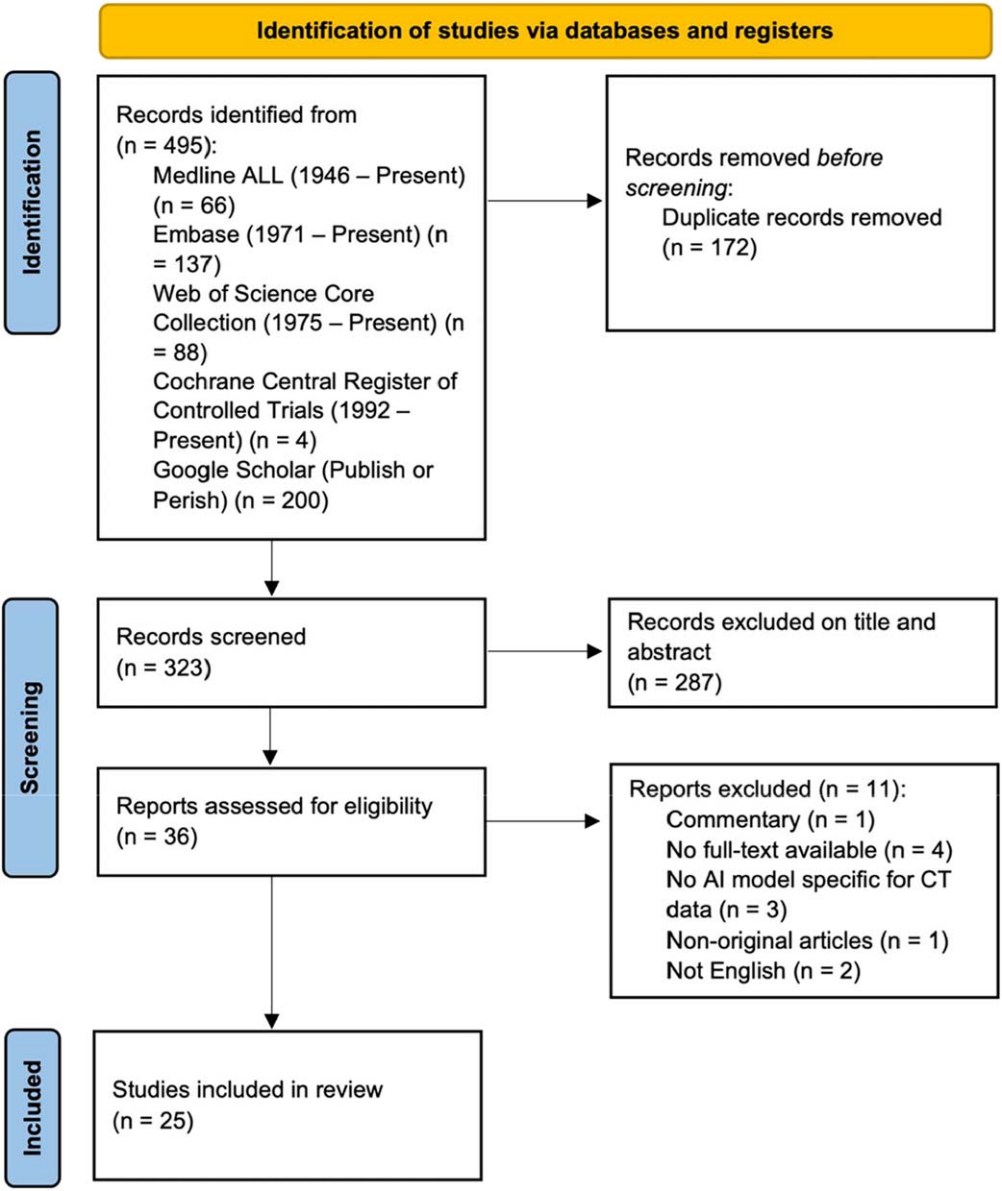
PRISMA flowchart of the study selection.

The search identified a total of 495 records. After removing duplicates, 323 records were screened on title and abstract. Thirty-six records were eligible for the full-text screening. This resulted in 25 studies meeting the inclusion criteria^14, 23-46^ (Fig. 1). Twenty studies were included in the meta-analysis^14,23,25,27–34,36–42,44,45^ and 5 were excluded because of a lack of data.^24,26,35,43,46^ The included studies scored between 4 and 8 points on the MINORS quality assessment (SDC 3, Supplemental Digital Content 1, http://links.lww.com/JTI/A334). Four studies were labeled as low-quality, with a score of 4 (23 to 26).

### Description of Studies

The number of rib fractures per study ranged from 256 (27) to 43,803 (28; Table 1). Nine studies used an external data set to test the model.^27–35^ Seventeen studies reported on the patients’ demographics.^14,27–29,31–34,36–44^ Fourteen reported about performing data selection.^14,27–30,32–34,36,38–40,42,44^ Thirteen studies used >1 CT scanner to acquire their data set, and a slice thickness range of 0.5 to 5.0 mm was reported.^28,30–34,36,39–42,44,45^ In 12 studies, the gold standard label assignment was performed by 2 radiologists.^14,27–30,32–34,36,37,41,43^ In 5 studies, the gold standard was established by more than 2 radiologists,^31,39,40,42,43^ and 1 study used only 1 radiologist.^45^ The other 3 studies used a radiologist and an orthopedic surgeon, 2 orthopedic surgeons, or 2 “attending doctors” to assign the gold standard label.^35,38,46^

**TABLE 1:** Literature Overview Including Studies Reporting on AI Models for Detection, Classification, and/or Segmentation of Rib Fractures

References	No. patients	No. fractures	Type of AI model	Distribution pt (train/val/test)	External test data set	Demographic report	Data selection	No. different CT scanners used	Thickness slice (mm)	Gold standard
Chen et al ^24^	120	N.R.	Detection	120//	N.R.	N.R.	N.R.	N.R.	N.R.	N.R.
Jin et al^39^	900	7473	Segmentation	720/60/120	N.R.	Yes	Yes	2	1.0-1.25	3 radiologists
Weikert et^41^	159	991	Detection/classification	N.R.	N.R.	Yes	N.R.	3	1.5	2 radiologists
Zhou et al ^44^	1,049	25,054	Detection/classification	876/173/	N.R.	Yes	Yes	4	1.0-5.0	2 radiologists
Castro-zunti et al ^37^	612	20,646	Detection/classification	428/122/62	N.R.	Yes	N.R.	1	2.0	2 radiologists
Hu et al ^46^	596	N.R.	Detection	389/117/88	N.R.	N.R.	N.R.	N.R.	5.0	N.R.
Kaiume et al^27^	39	256	Detection	3644†//39	Yes	Yes	Yes	1	0.625	2 radiologists
Meng et al ^29^	8,829	34,689	Detection/classification	6823/1706/300	Yes#	Yes	Yes	1	0.625	2 radiologists
Wu et al^31^	10,943	9590	Detection	2425//105§	Yes	Yes	N.R.	6	0.625-5.0	1 and 3 radiologists∥
Yao et al^14^	1,707	8271	Detection	1507/100/100	N.R.	Yes	Yes	N.R.	<2.0	2 radiologists
Zhang et al ^33^	3,580	15,947	Detection/classification	2855//198	Yes	Yes	Yes	3	0.625	2 radiologists
Azuma et al^36^	539	4906	Detection/classification	511//28	N.R.	Yes	Yes	4	<5.0	2 radiologists
Chai et al ^23^	N.R.	1135	Detection	224/151/310	N.R.	N.R.	N.R.	N.R.	N.R.	N.R.
Gao et al^45^	600	2868	Segmentation	2360/207/301*	N.R.	N.R.	N.R.	4	<2.0	1 radiologists
Inoue et al ^38^	200	1897	Detection	181//19	N.R.	Yes	Yes	N.R.	0.5-5.0	2 orthopedic surgeons
Niiya et al ^30^	918	N.R.	Detection/classification	897//21	Yes	N.R.	Yes	3	1.0	2 radiologists
Su et al ^25^	30	13,230‡	Detection	11,314/212/750‡	N.R.	N.R.	N.R.	N.R.	N.R.	N.R.
Wang et al ^28^	9265	43,803	Detection/classification	7869/420/976	Yes	Yes	Yes	5	0.625-5.0	2 radiologists
Yang et al ^32^	786	3562	Detection/classification	591//1,206¶	Yes	Yes	Yes	3	0.625-1.0	2 radiologists
Zhang et al ^42^	260	2345	Segmentation	200/60/	N.R.	Yes	Yes	2	1.0-1.25	3 radiologists
Zhou et al^34^	640	2853	Detection/classification	512//90	Yes	Yes	Yes	4	1.0-1.5	2 radiologists
Zhou et al ^43^	818	3134	Segmentation	572/82/164	N.R.	Yes	N.R.	1	1.25-5.0	3 radiologists
Edamadake et al ^35^	555	4746	Detection	378/42/135	Yes	No	N.R.	N.R.	1.0-1.25	5 radiologists + 2 radiologist and surgeon
Lin et al^40^	2100	N.R.	Detection	1440/360/300	No	Yes	Yes	3	0.625	4 radiologists
Wang et al^26^	500	N.R.	Detection	420//80	N.R.	N.R.	N.R.	N.R.	N.R.	N.R.

* Rib fractures, not patients.

† The commercially available model was trained on this data set.

‡ Images of rib fractures; multiple images can exist for one rib fracture.

§ For the detection, a data set of 105 patients was used.

∥ Number of radiologists depended on the test set used.

¶ This is cohort 2 for testing.

# No explanation of where the external data comes from.

N.R. indicates not reported.

### Performance of Models

#### Detection

For the detection of rib fractures, 43,834 patients from 21 studies were included^14, 23-38, 40-44, 46^ (SDC 4, Supplemental Digital Content 1, http://links.lww.com/JTI/A334). These studies reported sensitivities ranging from 0.645 (27) to 0.971 (44). The pooled proportion for sensitivity was 86.7% (95% CI: 82.6%-90.2%) and that of the clinicians was 75.4% (95% CI: 68.1%-82.1%; Table 2; SDC 7, Supplemental Digital Content 1, http://links.lww.com/JTI/A334). The lack of overlap between the 2 CIs implies that the DL model performs significantly better than clinicians in detecting rib fractures. The confidence intervals for sensitivity when comparing the external data set to clinicians show a pooled proportion of 86.6% (95% CI: 81.7%-90.8%) and 78.0% (95% CI: 68.9%-85.8%), respectively (Table 3; SDC 8, Supplemental Digital Content 1, http://links.lww.com/JTI/A334). These confidence intervals do overlap; the difference in performance between the detection DL model and clinicians remains undecided. Two studies did not mention the sensitivity of their model but reported mAP values of 42.3% (23) and 89.2% (25). When sorting the studies on highest sensitivity, there is no correlation between superior performance DL models and type of DL algorithm employed, MINORS score of the studies, or distribution of patients across the training, validating, and testing data set.

**TABLE 2:** Pooled Sensitivity of the Different DL Models and Clinicians

**Parameter**	**Studies (n)**	**Patients (n)**	**Cochran Q (P-value)**	***I*^2^ (95% CI)**	**Pooled proportion (95% CI)**
Detection model					
Sensitivity	15	12,350	531.4 (<0.001)	97% (96%-98%)	86.7% (82.6%-90.2%)
Clinician sensitivity	11	10,454	618.5 (<0.001)	98% (98%-99%)	75.4% (68.1%-82.1%)
Segmentation model					
Sensitivity	3	3093	18.0 (0.0001)	89% (69%-96%)	92.4% (88.9%-95.3%)
Classification models					
Displaced fractures					
Sensitivity	5	3933	20.3 (=0.0004)	80% (54%-92%)	97.3% (95.6%-98.5%)
Clinician Sensitivity	5	3933	25.5 (<0.001)	84% (65%-93%)	88.2% (84.8%-91.3%)
Nondisplaced fractures					
Sensitivity	6	2776	177.8 (<0.001)	97% (96%-98%)	77.4& (66.2%-86.9%)
Clinician sensitivity	5	2106	57.9 (<0.001)	93% (87%-96%)	71.7% (62.2%-80.3%)
Acute fractures					
Sensitivity	5	3563	133.6 (<0.001)	97% (95%-98%)	86.2% (76.9-93.4%)
Clinician sensitivity	4	2875	154.9 (<0.001)	98% (97%-99%)	78.9% (60.0-93.0%)
Old fractures					
Sensitivity	8	8539	495.5 (<0.001)	98% (98%-99%)	89.3% (81.8-95.0%)
Clinician sensitivity	7	8333	831.9 (<0.001)	99% (99%-99%)	80.2% (67.0-90.7%)

See SDC 7, Supplemental Digital Content 1, http://links.lww.com/JTI/A334 for forest and funnel plots.

N.A. indicates not available.

**TABLE 3:** Pooled Sensitivity of the Detection DL Models With External Validation

Parameter	Studies (n)	Patients (n)	Cochran Q (*P*-value)	*I*^2^ (95% CI)	Pooled proportion (95% CI)
Sensitivity	8	7685	184.2 (<0.001)	96% (94%-97%)	86.6% (81.7%-90.8%)
Clinician sensitivity	5	7067	237.9 (<0.001)	98% (97%-99%)	78.0% (68.9%-85.8%)

See SDC 8, Supplemental Digital Content 1, http://links.lww.com/JTI/A334 for forest and funnel plots.

N.A. indicates not available.

Eighteen studies reported either on FPs per scan or the F1-score, ranging from 0.14 (29) to 2.71 (36) and 0.652 (38) to 0.97 (35), respectively. The precision values ranged from 0.602 (38) to 0.96 (35), and the accuracy from 0.814 (24) to 0.96 (37). The reported mean with SD of clinicians’ sensitivity, precision, and F1-score were 0.74±0.12, 0.91±0.07, and 0.85±0.02, respectively.

Eleven out of 19 studies reported higher mean sensitivity for the DL model than for clinicians.^14,24,29,30,32,34,35,37,40,43,46^ One study showed a lower sensitivity for rib fracture detection using a DL model than clinicians, 0.794 versus 0.834 (95% CI: 80.7-85.8; 33).

#### Segmentation

For the segmentation of rib fractures, 2578 patients from 4 studies were included^39, 42, 43, 45^ (SDC 5, Supplemental Digital Content 1, http://links.lww.com/JTI/A334). These studies showed sensitivities ranging from 0.813 (43) to 0.95 (42). The pooled proportion was 92.4% (95% CI: 88.9%-95.3%), and there was no comparison with clinicians (Table 2; SDC 7, Supplemental Digital Content 1, http://links.lww.com/JTI/A334). Three studies reported the DSC and IoU,^39,42,45^ ranging from 0.628 (42) to 0.854 (45) and 0.488 (42) to 0.804 (45), respectively. Zhou et al^43^ described the outcome as “not high” in the absence of further quantification. One study reported a specificity and accuracy of 0.876 and 0.881, respectively.^45^ Jin et al^39^ compared their DL model results with those of clinicians who achieved a sensitivity, DSC, and IoU of 0.831, 0.647, and 0.478, respectively. This is lower than their DL model, with a sensitivity, DSC, and IoU of 0.920, 0.751, and 0.556, respectively.

#### Classification

For the classification of rib fractures, 26,359 patients from ten studies were included (28 to 30, 32 to 34, 36, 37, 41, 44; SDC 6, Supplemental Digital Content 1, http://links.lww.com/JTI/A334). Cumulative labels that were described in various studies were “old fracture,” “acute fracture,” healing, displaced, nondisplaced, and buckle.

Four studies reported the classification of acute fractures with sensitivities ranging from 0.677 (41) to 0.947 (30).^30,34,37,41,44^ Eight studies reported old fractures, with sensitivities ranging from 0.587 (41) to 0.968 (34).^28,29,32–34,37,41,44^ Displaced fractures were classified in 5 studies,^28,29,32,33,36^ with a sensitivity ranging from 0.924^33^ to 0.995 (36). Six studies reported nondisplaced fractures,^28,29,32,33,36,41^ with sensitivity ranging from 0.732 (36) to 0.853 (28). Meng et al^28–30,33^ classified buckle fractures with sensitivities ranging from 0.581 (33) to 0.896 (30).

For clinicians, the sensitivity for classifying acute fractures ranged from 0.677 (33) to 0.98 (37), old fractures from 0.533 (44) to 0.943 (33), displaced fractures from 0.83 (36) to 0.934 (33), nondisplaced fractures from 0.35 (36) to 0.849 (29), and buckle fractures from 0.554 (28) to 0.756 (29). Zhou et al^34,44^ reported acute, old, and healing fractures. Healing fractures were defined as those more than 3 weeks old. The sensitivities for classifying healing fractures were 1.000 (34) and 0.859 (44), and for the clinicians, 0.751 (34) and 0.614 (44).

The pooled proportions for the sensitivity for the classification of displaced rib fractures for both the DL models and clinicians were 97.3% (95% CI: 95.6%-98.5%) and 88.2% (95% CI: 84.8%-91.3%), respectively. Thus, DL models performed significantly better. Nondisplaced fractures had sensitivities of the DL models of 77.4% (95% CI: 66.2%-86.9%) and of the clinicians of 71.7% (95% CI: 62.2%-80.3%). Acute fractures had a pooled sensitivity of 86.2% (95% CI: 76.9%-93.4%) and 78.9% (95% CI: 60.0%-93.0%) for DL models and clinicians, respectively. For old fractures, the sensitivities of DL models were 89.3% (95% CI: 81.8%-95.0%) and of the clinicians were 80.2 (95% CI: 67.0%-90.7%; Table 2; SDC 7, Supplemental Digital Content 1, http://links.lww.com/JTI/A334). Because the CIs overlap, the difference between the DL models and clinicians for nondisplaced, acute, and old fractures is better, but undecided.

## DISCUSSION

This review evaluates the performance of DL models in the detection, segmention, and classification of rib fractures using CT data. Based on the included studies, most DL models have higher sensitivities than clinicians for the detection, segmentation, and classification of rib fractures.

The majority of studies reported detection either as a primary outcome or secondary to classification. In daily practice, the high number of missed fractures may have negative clinical consequences, such as inadequate pain management and pulmonary complications.^1,6^ Future research will have to show if this shortcoming could lead to undertreatment of the patient, with potential negative consequences, and if DL models might be helpful in overcoming this.

The delineation of rib fractures is technically challenging because the fracture parts can vary in size, position, and orientation. This may be the reason why a segmentation model cannot perform this task perfectly. None of the studies that reported segmentation used an external test set to validate the results. Possible explanations might be the lower clinical added value, the higher computational power required, or the labor-intensive process of creating a suitable data set for segmentation.

Several studies have reported on the classification of rib fractures.^28–30,32–34,36,37,41,44^ Unfortunately, there was no uniform definition of the different fracture types among the included studies. This could be a reason why the pooled proportions of the sensitivity for the DL models were not significantly higher than those for the clinicians across different rib fracture types, except for displaced fractures. Recently, efforts have been made by the Chest Wall Injury Society (CWIS) to standardize the classification of rib fractures. The CWIS taxonomy defines fractures based on their location, degree of displacement, fracture type, and association with other fractures based on the Delphi consensus.^15^ This might be a starting point to uniformly define fracture types. However, future studies must focus on the accuracy of DL models while using this Delphi-based classification system.

In 5 studies, a tradeoff between sensitivity and FP rate was achieved by setting a threshold.^27,30,35,36,44^ The studies in this review showed a preference for high sensitivity, while accepting a higher FP rate. This seems reasonable from a clinical perspective to prevent undertreatment.

Thus, DL models have the potential to improve current practice due to their high sensitivity, but clinicians have to interpret DL outcomes cautiously because various shortcomings still exist. High FP rates might result in overtreatment, with consequent increase in health care costs. Implementation studies must evaluate this socioeconomic risk.

DL models are highly dependent on the data used for training purposes and tend to perform better with homogeneous data sets.^47,48^ This is also shown in the nonsignificantly different pooled proportions of sensitivity for the detection DL models using external data sets compared with clinicians. Real-world (external) data sets are heterogeneous because of factors such as differences in CT scanners, slice thicknesses, imaging protocols, and populations. A large number of studies have not reported this information in their data sets. The lack of transparency can limit the reproducibility of such studies and hinder the generalizability of the findings. Collaborations between institutions interested in both DL models and patient care might overcome this knowledge gap while testing shared patient data.

In addition, while most studies used 2 or more clinicians to define the gold standard, it is important to maintain a critical attitude toward the results. If the gold standard and the ground-truth for the DL models were assessed by the same clinicians, this could lead to confirmation bias. In most studies, these were assessed by different clinicians. Furthermore, it is difficult to determine the accuracy of the gold standard and whether this mimics daily practice. The assignment of the gold standard was done by radiologists and other clinicians with varying years of experience in assessing radiographs, and should be taken into consideration when interpreting the gold standard. The vulnerability of the gold standard is indicated by the high frequency of missed fractures in clinical practice and limited interobserver agreement. The findings in this study suggest that DL models can be helpful to come to more consistent conclusions in the near future compared with current practice.

A shared thought in the field is that DL model performance improves with increasing amounts of data.^49^ However, this trend was not evident in this review. This might indicate that the data sets were homogeneous and that overfitting was possible. Nevertheless, three out of 5 studies showing the highest sensitivities used >1 CT scanner^30,32,44^ and 4 out of 5 used an external test set,^29,30,32,35^ indicating heterogeneous data input. One study showed that a larger data set resulted in an increase in performance of ~4%.^30^ These authors used the model of Azuma et al^36^ and performed training with an additional 333 CT scans. This increased the sensitivity from 89.4% to 93.5%, with a decreased FP per case from 2.5 to 1.9. However, the authors did not report on the data set characteristics, used a small test set, and excluded data with confusing artifacts. Thus, in this literature review, the performance of the DL models cannot be directly related to the data sets used.

This study has some limitations that need to be acknowledged. First, not every study reported the same outcomes, making part of the intended pooled analysis impossible. Furthermore, the pooled results should be interpreted with caution owing to the large heterogeneity across the studies, and several studies had to be excluded based on a lack of reported outcomes of interest. Furthermore, other factors associated with the usefulness of DL models, such as time efficiency and technical specifications, have not been addressed. Although sensitivity was reported by all studies except 2,^23,25^ no uniform measure was used for the FPs, if mentioned at all. Studies have used FP ratios per scan and F1-scores, making it difficult to quantitatively compare the performance of models using these metrics. The definition of an FP remains unclear in several studies. This makes the comparison between the results of these studies difficult and raises the need for a unanimous way of presenting results in AI studies. Future research should aim to expand the clinical added value of DL models by focusing on more advanced classification and prediction models that could provide important information for patient management and treatment planning. With further advancements in DL and increased collaboration between researchers and clinicians, DL models have the potential to become indispensable tools for the diagnosis and management of rib fractures. After implementation, socioeconomic consequences must be evaluated carefully.

In conclusion, DL models can detect, segment, and classify rib fractures and have shown significant improvements over the last few years. DL models outperform clinicians in rib fracture detection and classification of displaced rib fractures. Fewer data are available regarding the evaluation of segmentation models. The currently available data lacks validation on external test sets.

## Supplementary Material

SUPPLEMENTARY MATERIAL
