# Supplementary material for: Detection, Classification, and Segmentation of Rib Fractures From CT Data Using Deep Learning Models: A Review of Literature and Pooled Analysis
Source: J Thorac Imaging. 2025 May 23;40(5):e0833. doi: 10.1097/RTI.0000000000000833 (PMC12369507; doi:10.1097/RTI.0000000000000833)
Supplement: SUPPLEMENTARY MATERIAL [file rti-40-e0833-s001.docx]

**Supplemental Digital Content (SDC) 1: Search strategy**

**Medline**

("Rib Fractures"/ OR ("Ribs"/ AND "Fractures, Bone"/) OR (((rib* OR costal OR costa) ADJ6 (fractur* OR broken)) OR flail-chest*).ab,ti,kf.) AND (exp "Machine Learning"/ OR exp "Artificial Intelligence"/ OR (((machine* OR deep) ADJ3 (learning*)) OR ((neural) ADJ3 (network*)) OR ((classification*) ADJ3 (algor* OR binar* OR multi* OR automat*)) OR classifier* OR ((feature* OR automat*) ADJ3 (detection*)) OR ((data* OR comput*) ADJ3 (algo*)) OR ((artificial* OR machine) ADJ3 (intelligen*)) OR AI OR ((automat*) ADJ3 (reason*)) OR segmentation* OR bayes* OR ((feature*) ADJ3 (extract* OR detect*)) OR fuzzy).ab,ti,kf.)

**Embase**

('rib fracture'/exp OR (rib/de AND fracture/de) OR (((rib* OR costal OR costa) NEAR/6 (fractur* OR broken)) OR flail-chest*):ab,ti,kw) AND ('machine learning'/exp OR 'segmentation algorithm'/de OR 'artificial intelligence'/exp OR 'image segmentation'/exp OR (((machine* OR deep) NEAR/3 (learning*)) OR ((neural) NEAR/3 (network*)) OR ((classification*) NEAR/3 (algor* OR binar* OR multi* OR automat*)) OR classifier* OR ((feature* OR automat*) NEAR/3 (detection*)) OR ((data* OR comput*) NEAR/3 (algo*)) OR ((artificial* OR machine) NEAR/3 (intelligen*)) OR AI OR ((automat*) NEAR/3 (reason*)) OR segmentation* OR bayes* OR ((feature*) NEAR/3 (extract* OR detect*)) OR fuzzy):ab,ti,kw)

**Web of Science**

TS=((((rib* OR costal OR costa) NEAR/5 (fractur* OR broken)) OR flail NEXT/ chest*) AND (((machine* OR deep) NEAR/2 (learning*)) OR ((neural) NEAR/2 (network*)) OR ((classification*) NEAR/2 (algor* OR binar* OR multi* OR automat*)) OR classifier* OR ((feature* OR automat*) NEAR/2 (detection*)) OR ((data* OR comput*) NEAR/2 (algo*)) OR ((artificial* OR machine) NEAR/2 (intelligen*)) OR AI OR ((automat*) NEAR/2 (reason*)) OR segmentation* OR bayes* OR ((feature*) NEAR/2 (extract* OR detect*)) OR fuzzy))

**Cochrane CENTRAL**

((((rib* OR costal OR costa) NEAR/6 (fractur* OR broken)) OR flail NEXT/1 chest*):ab,ti,kw) AND ((((machine* OR deep) NEAR/3 (learning*)) OR ((neural) NEAR/3 (network*)) OR ((classification*) NEAR/3 (algor* OR binar* OR multi* OR automat*)) OR classifier* OR ((feature* OR automat*) NEAR/3 (detection*)) OR ((data* OR comput*) NEAR/3 (algo*)) OR ((artificial* OR machine) NEAR/3 (intelligen*)) OR AI OR ((automat*) NEAR/3 (reason*)) OR segmentation* OR bayes* OR ((feature*) NEAR/3 (extract* OR detect*)) OR fuzzy):ab,ti,kw)

**Google Scholar**

"machine learning"|"artificial intelligence"|AI rib|ribs fractures|fracture CT|"computed tomography"

**SDC 2: Formulas**

$sensitivity= \frac{TP}{TP+FN}$

$precision= \frac{TP}{TP+FP}$

$F1_{score}= 2*\frac{sensitivity*precision}{sensitivity+precision}$

$accuracy= \frac{TP+TN}{TP+FP+TN+FN}$

$mAP= \frac{1}{|classes|}\sum_{c\in classes} \frac{\#TP(c)}{\#TP(c)+\#FP(c)}$

$specificity= \frac{TN}{TN+FP}$

$DSC= 2* \frac{|X \cap Y|}{|X| + |Y|}$

$IoU= \frac{|X \cap Y|}{|X| \cup|Y|}$

TP, true positives; FN, false negatives; TN, true negatives; FP, false positives; mAP, mean average precision; X, ground truth segmentation; Y, model output segmentation; $\cap$, intersection; ∪, union; DSC and IoU, are pixel based measures.

**SDC 3: Quality assessment according to the modified MINORS criteria**

| **Author (year)** | **Study aim** | **Data selection/input features** | **(Type of) AI model** | **External test dataset** | **Ground truth** | **Demographic report** | **Dataset distribution** | **Adequate statistical analyses** | **Total score** |
| --- | --- | --- | --- | --- | --- | --- | --- | --- | --- |
| **Chen *et al*. (2018) [24]** | 1 | 0 | 1 | 0 | 0 | 0 | 1 | 1 | **4** |
| **Jin *et al*. (2020) [39]** | 1 | 1 | 1 | 0 | 1 | 1 | 1 | 1 | **7** |
| **Weikert *et al*. (2020) [41]** | 1 | 0 | 1 | 0 | 1 | 1 | 1 | 1 | **6** |
| **Zhou *et al*. (2020) [44]** | 1 | 1 | 1 | 0 | 1 | 1 | 1 | 1 | **7** |
| **Castro-zunti *et al*. (2021) [37]** | 1 | 0 | 1 | 0 | 1 | 1 | 1 | 1 | **6** |
| **Hu *et al*. (2021) [46]** | 1 | 0 | 1 | 0 | 1 | 0 | 1 | 1 | **5** |
| **Kaiume *et al*. (2021) [27]** | 1 | 1 | 1 | 1 | 1 | 1 | 1 | 1 | **8** |
| **Meng *et al*. (2021) [29]** | 1 | 1 | 1 | 1 | 1 | 1 | 1 | 1 | **8** |
| **Wu *et al*. (2021) [31]** | 1 | 0 | 1 | 1 | 1 | 1 | 1 | 1 | **7** |
| **Yao *et al*. (2021) [14]** | 1 | 1 | 1 | 0 | 1 | 1 | 1 | 1 | **7** |
| **Zhang *et al*. (2021) [33]** | 1 | 1 | 1 | 1 | 1 | 1 | 1 | 1 | **8** |
| **Azuma *et al*. (2022) [36]** | 1 | 1 | 1 | 0 | 1 | 1 | 1 | 1 | **7** |
| **Chai *et al*. (2022) [23]** | 1 | 0 | 1 | 0 | 0 | 0 | 1 | 1 | **4** |
| **Gao *et al*. (2022) [45]** | 1 | 0 | 1 | 0 | 1 | 0 | 1 | 1 | **5** |
| **Inoue *et al*. (2022) [38]** | 1 | 1 | 1 | 0 | 1 | 1 | 1 | 1 | **7** |
| **Niiya *et al*. (2022) [30]** | 1 | 1 | 1 | 1 | 1 | 0 | 1 | 1 | **7** |
| **Su *et al*. (2022) [25]** | 1 | 0 | 1 | 0 | 0 | 0 | 1 | 1 | **4** |
| **Wang *et al*. (2022) [28]** | 1 | 1 | 1 | 1 | 1 | 1 | 1 | 1 | **8** |
| **Yang *et al*. (2022) [32]** | 1 | 1 | 1 | 1 | 1 | 1 | 1 | 1 | **8** |
| **Zhang *et al*. (2022) [42]** | 1 | 1 | 1 | 0 | 1 | 1 | 1 | 1 | **7** |
| **Zhou *et al*. (2022) [34]** | 1 | 1 | 1 | 1 | 1 | 1 | 1 | 1 | **8** |
| **Zhou *et al*. (2022) [43]** | 1 | 0 | 1 | 0 | 1 | 1 | 1 | 1 | **6** |
| **Edamadake *et al*. (2023) [27]** | 1 | 0 | 1 | 1 | 1 | 0 | 1 | 1 | **6** |
| **Lin *et al*. (2023) [40]** | 1 | 1 | 1 | 0 | 1 | 1 | 1 | 1 | **7** |
| **Wang *et al*. (2023) [26]** | 1 | 0 | 1 | 0 | 0 | 0 | 1 | 1 | **4** |

**SDC 4: Results of the detection models for rib fractures**

| **Author (year)** | **Sensitivity** | **Precision** | **FP/scan** | **F1-score** | **Accuracy** | **mAP** | **Clinician sensitivity** | **Clinician precision** | **Clinican F1-score** | **Clinician accuracy** | **Clinician FP/scan** |
| --- | --- | --- | --- | --- | --- | --- | --- | --- | --- | --- | --- |
| **Chen *et al*. (2018) [24]** | 0.904 | N.R. | N.R. | N.R. | 0.814 | N.R. | N.R. | N.R. | N.R. | N.R. | N.R. |
| **Weikert *et al*. (2020) [41]^1^** | 0.657 | N.R. | 0.16 | N.R. | N.R. | N.R. | N.R. | N.R. | N.R. | N.R. | N.R. |
| **Zhou *et al*. (2020) [44]** | 0.832 | 0.824 | 1.1 | 0.825 | N.R. | N.R. | 0.624 | 0.803 | N.R. | N.R. | N.R. |
| **Castro-zunti *et al*. (2021) [37]** | 0.94 | N.R. | N.R. | N.R. | 0.96 | N.R. | 0.92 | N.R. | N.R. | 0.92 | N.R. |
| **Hu *et al*. (2021) [46]** | 0.909 | 0.69 | N.R. | 0.784 | 0.825 | N.R. | 0.807 | 1 | 0.893 | 0.93 | N.R. |
| **Kaiume *et al*. (2021) [27]** | 0.645 | N.R. | 1.1 | 0.711 | N.R. | N.R. | * N.R. | N.R. | N.R. | N.R. | N.R. |
| **Meng *et al*. (2021) [29]** | 0.922 | 0.949 | 0.14 | 0.94 | 0.863 | N.R. | 0.812 | 0.885 | 0.845 | N.R. | N.R. |
| **Wu *et al*. (2021) [31]^2^** | 0.849 | 0.822 | 0.764 | 0.833 | N.R. | N.R. | 0.797 | 0.906 | 0.841 | N.R. | 0.448 |
| **Yao *et al*. (2021) [14]** | 0.913 | 0.869 | N.R. | 0.89 | N.R. | N.R. | 0.773 | 0.932 | 0.843 | N.R. | N.R. |
| **Zhang *et al*. (2021) [33]** | 0.794 | N.R. | 0.43 | N.R. | N.R. | N.R. | 0.834 | N.R. | N.R. | N.R. | 0.18 |
| **Azuma *et al*. (2022) [36]** | 0.837 | N.R. | 2.71 | N.R. | N.R. | N.R. | 0.51 | N.R. | N.R. | N.R. | 0.03 |
| **Chai *et al*. (2022) [23]** | N.R. | N.R. | N.R. | N.R. | N.R. | 0.423 | N.R. | N.R. | N.R. | N.R. | N.R. |
| **Inoue *et al*. (2022) [38]** | 0.713 | 0.602 | N.R. | 0.652 | N.R. | N.R. | 0.609 | 0.949 | N.R. | N.R. | N.R. |
| **Niiya *et al*. (2022) [30]** | 0.935 | N.R. | 1.9 | N.R. | N.R. | N.R. | N.R. | N.R. | N.R. | N.R. | N.R. |
| **Su *et al*. (2022) [25]** | N.R. | N.R. | N.R. | N.R. | N.R. | 0.892 | N.R. | N.R. | N.R. | N.R. | N.R. |
| **Wang *et al*. (2022) [28]** | 0.85 | N.R. | 0.35 | N.R. | N.R. | N.R. | 0.65 | N.R. | N.R. | N.R. | 0.32 |
| **Yang *et al*. (2022) [32]^2^** | 0.922 | N.R. | N.R. | 0.827 | N.R. | N.R. | 0.795 | N.R. | 0.839 | N.R. | N.R. |
| **Zhou *et al*. (2022) [34]** | Ri 0.9711 | N.R. | 0.17 | N.R. | N.R. | N.R. | N.R. | N.R. | N.R. | N.R. | N.R. |
| **Zhou *et al*. (2022) [34]** | Le 0.9487 | N.R. | 0.17 | N.R. | N.R. | N.R. | N.R. | N.R. | N.R. | N.R. | N.R. |
| **Edamadake *et al*. (2023) [27]** | 0.97 | 0.96 | 2.24 | 0.97 | N.R. | N.R. | N.R. | N.R. | N.R. | N.R. | N.R. |
| **Lin *et al*. (2023) [40]** | 0.919 | N.R. | 0.069 | 0.92 | 0.956 | N.R. | 0.838 | N.R. | N.R. | 0.932 | N.R. |
| **Wang *et al*. (2023) [26]** | 0.817 | 0.82 | N.R. | 0.819 | N.R. | N.R. | N.R. | N.R. | N.R. | N.R. | N.R. |

FP, false positive; mAP, mean Average Precision; * Only compared with interns; ^1^ Based on per-finding level, not per-examination; ^2^On test set 2; N.R., not reported.

**SDC 5: Results of the segmentation models for rib fractures**

| **Author, year** | **Sensitivity** | **Specificity** | **FP/scan** | **IoU** | **DSC** | **Accuracy** | **Clinician sensitivity** | **Clinician specificity** | **Clinician Fp/scan** | **Clinician IoU** | **Clinician DSC** | **Clinician accuracy** |
| --- | --- | --- | --- | --- | --- | --- | --- | --- | --- | --- | --- | --- |
| **Jin *et al*. (2020) [39]** | 0.920 | N.R. | 5.27 | 0.556 | 0.715 | N.R. | 0.831 | N.R. | 1.80 | 0.478 | 0.647 | N.R. |
| **Gao *et al*. (2022) [45]** | 0.893 | 0.876 | N.R. | 0.804 | 0.854 | 0.881 | N.R. | N.R. | N.R. | N.R. | N.R. | N.R. |
| **Zhang *et al*. (2022) [42]** | 0.95 | N.R. | N.R. | 0.4881 | 0.6280 | N.R. | N.R. | N.R. | N.R. | N.R. | N.R. | N.R. |
| **Zhou *et al*. (2022) [43]** | 0.813 | N.R. | 3.1 | N.R. | N.R. | N.R. | N.R. | N.R. | N.R. | N.R. | N.R. | N.R. |

FP, false positives; IoU, Intersection-over-Union; DSC, Dice similarity coefficient; N.R., not reported.

**SDC 6: Results of the classification models for rib fractures**

| **Author (year)** | **Classes of different fracture types** | **Sensitivity** | | **Precision** | **F1-score** | **Accuracy** | **Clinician sensitivity** | **Clinician precision** | **Clinician F1-score** | **Clinician FP/scan** | **Clinician accuracy** |
| --- | --- | --- | --- | --- | --- | --- | --- | --- | --- | --- | --- |
| **Weikert *et al*. (2020) [41]** | Acute | 0.677 | N.R. | | N.R. | N.R. | N.R. | N.R. | N.R. | N.R. | N.R. |
|  | Old | 0.587 | N.R. | | N.R. | N.R. | N.R. | N.R. | N.R. | N.R. | N.R. |
|  | Non-displaced | 0.584 | N.R. | | N.R. | N.R. | N.R. | N.R. | N.R. | N.R. | N.R. |
| **Zhou *et al*. (2020) [44]** | Acute | 0.861 | 0.841 | | 0.849 | N.R. | 0.725 | 0.870 | N.R. | N.R. | N.R. |
|  | Healing | 0.859 | 0.857 | | 0.856 | N.R. | 0.614 | 0.848 | N.R. | N.R. | N.R. |
|  | Old | 0.775 | 0.774 | | 0.770 | N.R. | 0.533 | 0.692 | N.R. | N.R. | N.R. |
| **Castro-zunti *et al*. (2021) [37]** | Acute | 0.900 | N.R. | | N.R. | 0.900 | 0.980 | N.R. | N.R. | N.R. | 0.98 |
|  | Old | 0.960 | N.R. | | N.R. | 0.930 | 0.810 | N.R. | N.R. | N.R. | 0.81 |
|  | Non-fractured | 0.990 | N.R. | | N.R. | 0.990 | 0.930 | N.R. | N.R. | N.R. | 0.96 |
| **Meng *et al*. (2021) [29]** | Displaced | 0.983 | 0.909 | | N.R. | N.R. | 0.896 | 0.884 | N.R. | N.R. | N.R. |
|  | Non-displaced | 0.822 | 0.770 | | N.R. | N.R. | 0.849 | 0.712 | N.R. | N.R. | N.R. |
|  | Buckle | 0.826 | 0.879 | | N.R. | N.R. | 0.756 | 0.842 | N.R. | N.R. | N.R. |
|  | Old | 0.832 | 0.897 | | N.R. | N.R. | 0.889 | 0.970 | N.R. | N.R. | N.R. |
| **Zhang *et al*. (2021) [33]** | Displaced | 0.924 | N.R. | | N.R. | N.R. | 0.934 | N.R. | N.R. | N.R. | N.R. |
|  | Non-displaced | 0.783 | N.R. | | N.R. | N.R. | 0.803 | N.R. | N.R. | N.R. | N.R. |
|  | Buckle | 0.581 | N.R. | | N.R. | N.R. | 0.704 | N.R. | N.R. | N.R. | N.R. |
|  | Old | 0.965 | N.R. | | N.R. | N.R. | 0.943 | N.R. | N.R. | N.R. | N.R. |
| **Azuma *et al*. (2022) [36]** | Displaced | 0.995 | N.R. | | N.R. | N.R. | 0.830 | N.R. | N.R. | 0.03 | N.R. |
|  | Non-displaced | 0.732 | N.R. | | N.R. | N.R. | 0.350 | N.R. | N.R. | 0.03 | N.R. |
| **Niiya *et al*. (2022) [30]** | Acute | 0.947 | N.R. | | N.R. | N.R. | N.R. | N.R. | N.R. | N.R. | N.R. |
|  | Buckle | 0.896 | N.R. | | N.R. | N.R. | N.R. | N.R. | N.R. | N.R. | N.R. |
| **Wang *et al*. (2022) [28]** | Displaced | 0.984 | N.R. | | N.R. | N.R. | 0.885 | N.R. | N.R. | N.R. | N.R. |
|  | Non-displaced | 0.853 | N.R. | | N.R. | N.R. | 0.673 | N.R. | N.R. | N.R. | N.R. |
|  | Buckle | 0.704 | N.R. | | N.R. | N.R. | 0.554 | N.R. | N.R. | N.R. | N.R. |
|  | Old | 0.920 | N.R. | | N.R. | N.R. | 0.710 | N.R. | N.R. | N.R. | N.R. |
| **Yang *et al*. (2022) [32]^1^** | Non-displaced | 0.838 | N.R. | | 0.776 | N.R. | 0.740 | N.R. | 0.7755 | N.R. | N.R. |
|  | Displaced | 0.975 | N.R. | | 0.885 | N.R. | 0.848 | N.R. | 0.8686 | N.R. | N.R. |
|  | Old | 0.954 | N.R. | | 0.791 | N.R. | 0.896 | N.R. | 0.9082 | N.R. | N.R. |
|  | Old with distortion | 0.908 | N.R. | | 0.851 | N.R. | 0.714 | N.R. | 0.7937 | N.R. | N.R. |
| **Zhou *et al*. (2022) [34]** | Acute | 0.924 | 0.826 | | 0.872 | N.R. | 0.677 | 0.929 | 0.779 | N.R. | N.R. |
|  | Healing | 1.000 | 0.700 | | 0.824 | N.R. | 0.751 | 0.883 | 0.807 | N.R. | N.R. |
|  | Old | 0.968 | 0.909 | | 0.938 | N.R. | 0.781 | 0.809 | 0.790 | N.R. | N.R. |

^1^ On cohort 2; N.R., not reported.

**SDC 7: Forest and Funnel plots of pooled sensitivity of different DL models and clinicians**

|  | **Funnel plot** | **Forest plot** |
| --- | --- | --- |
| **Detection model** | 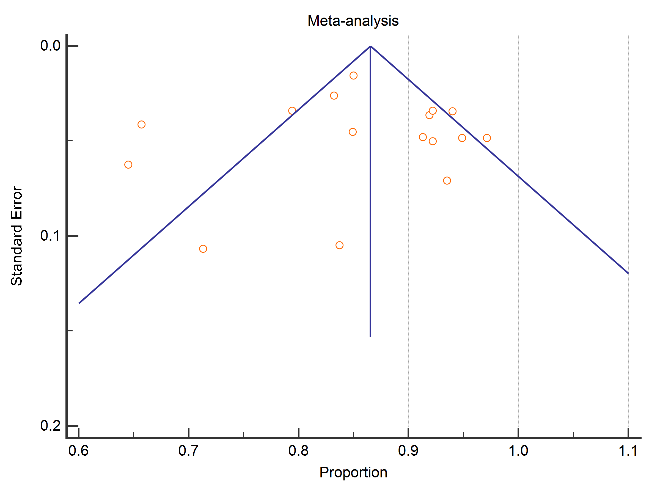 | 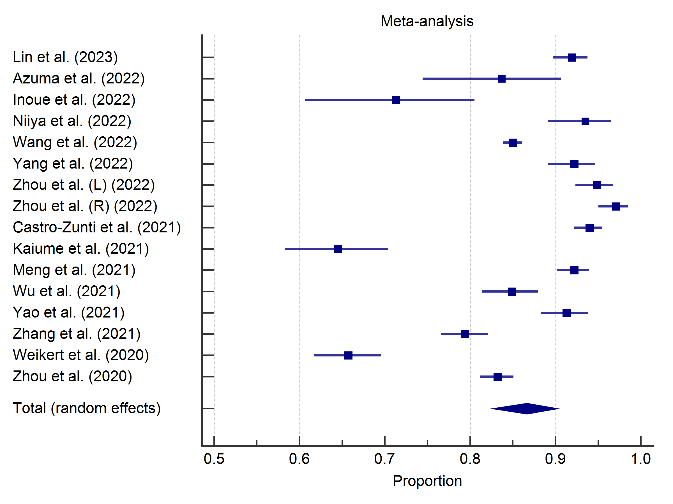 |
| **Clinicians detection model** | 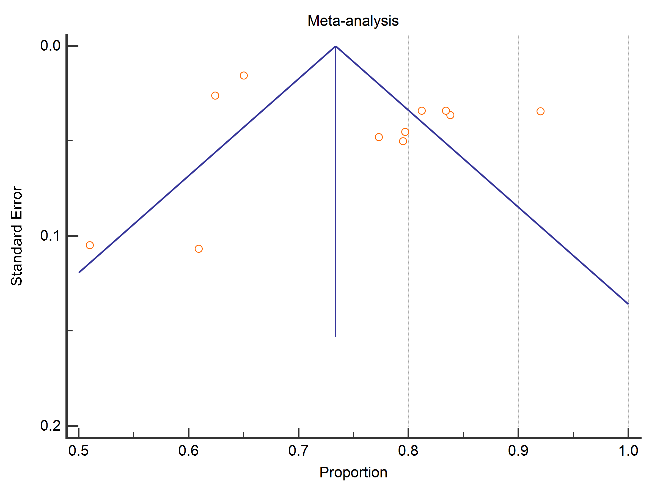 | 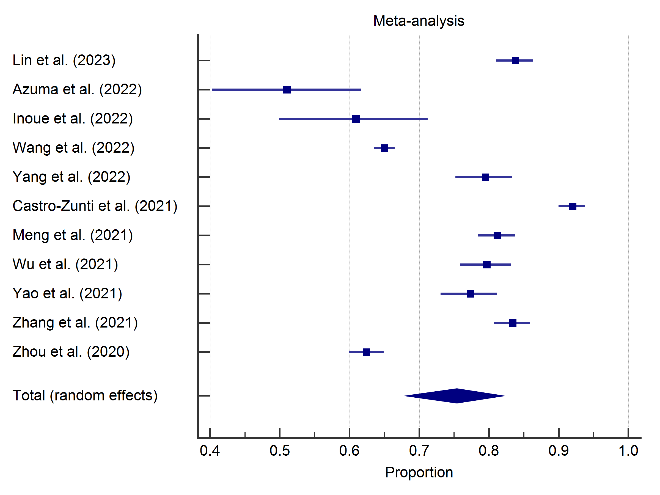 |
| **Segmentation model** | 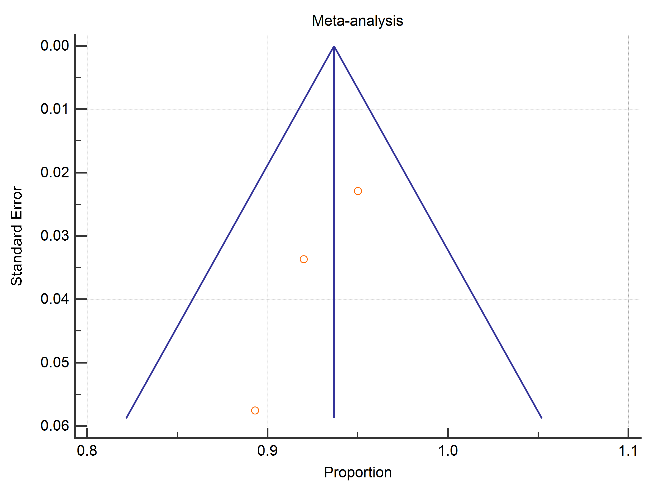 | 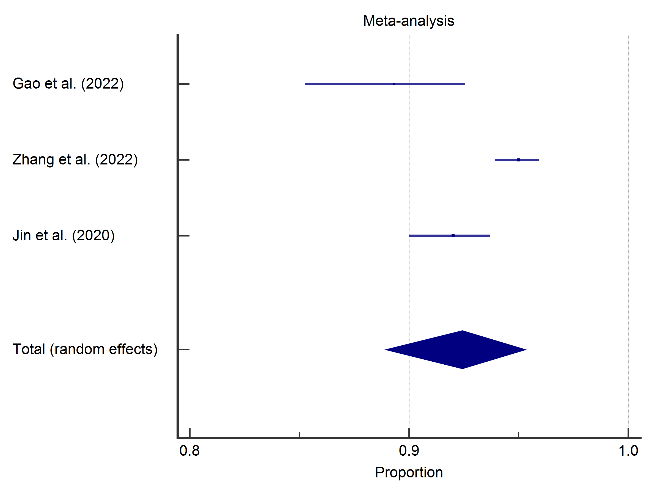 |

| **Classification models** | | |
| --- | --- | --- |
| Displaced fractures | 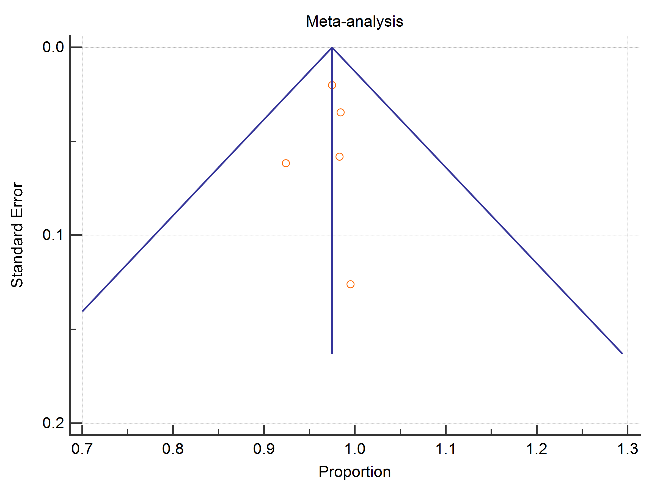 | 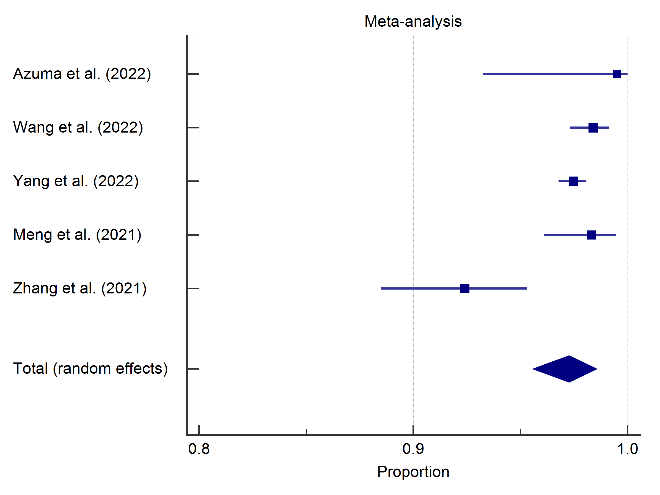 |
| Clinicians displaced fractures | 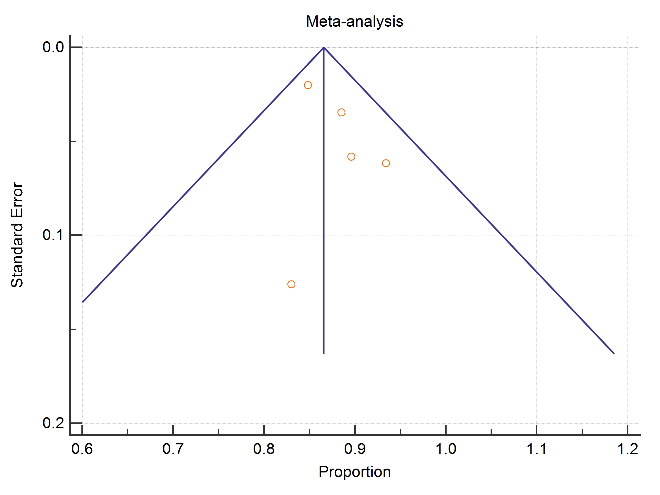 | 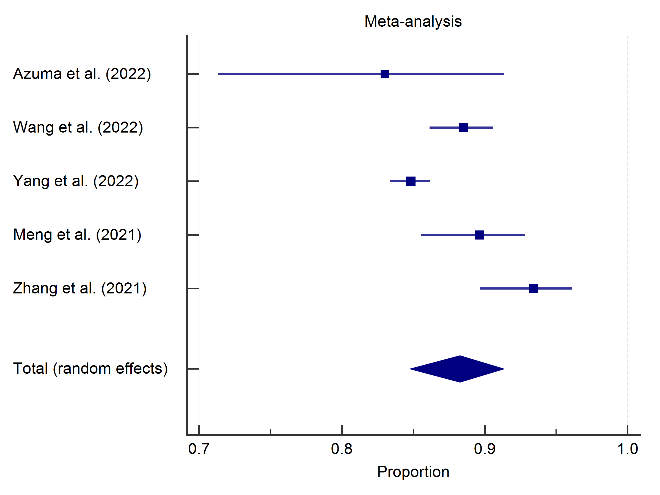 |
| Non-displaced fractures | 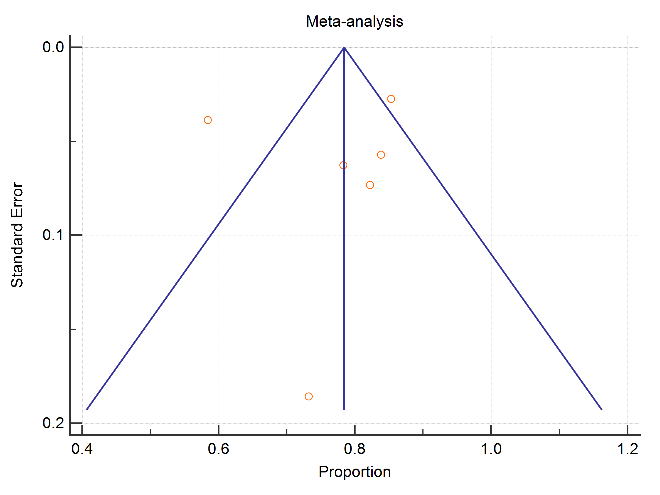 | 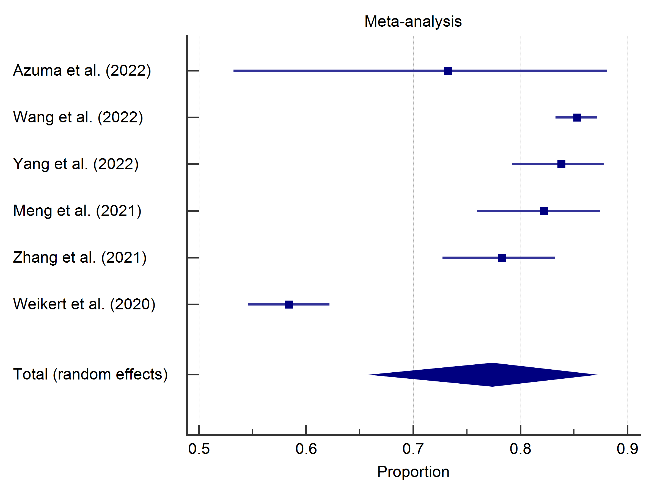 |
| Clinicians non-displaced fractures | 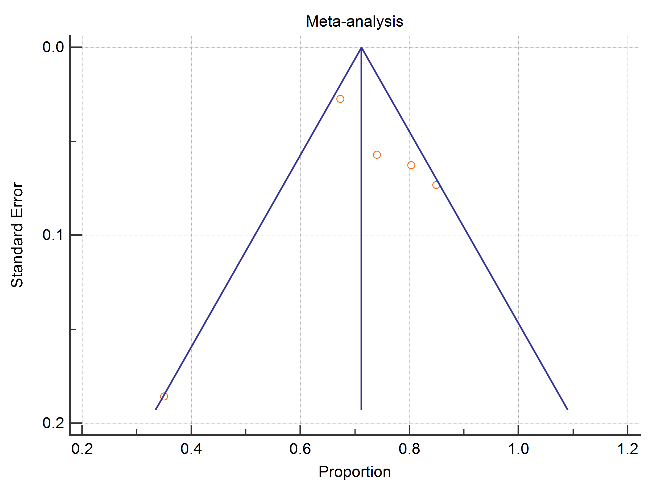 | 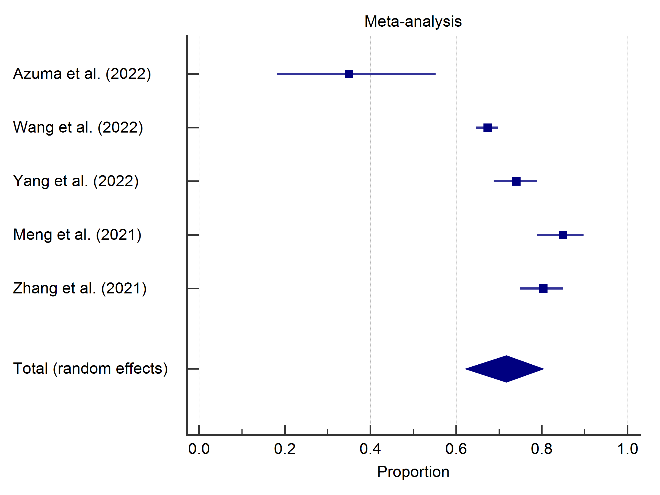 |
| Acute fractures | 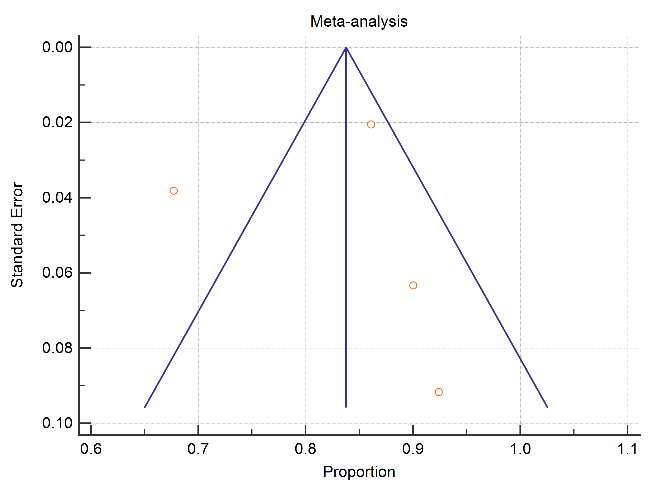 | 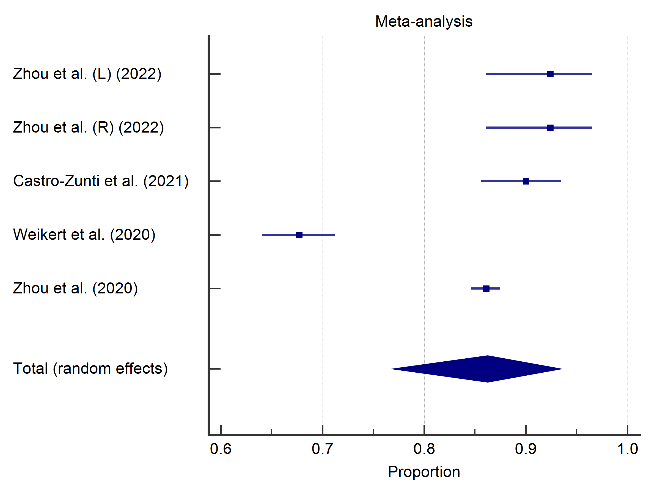 |
| Clinicians acute fractures | 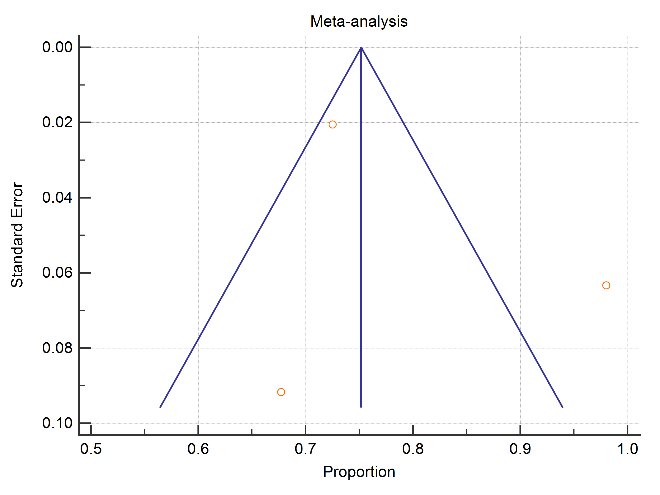 | 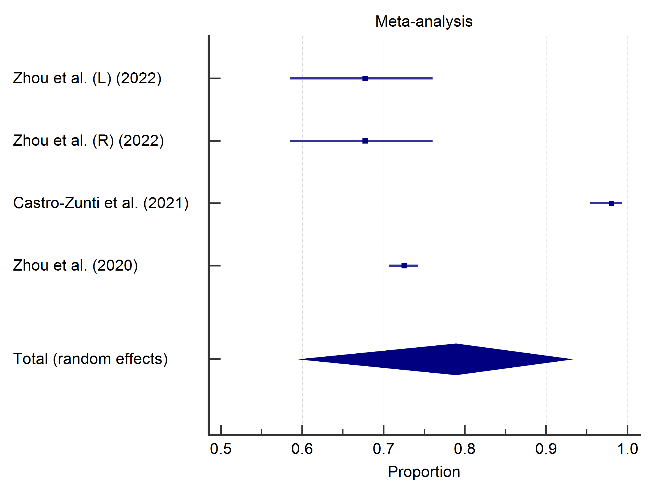 |
| Old fractures | 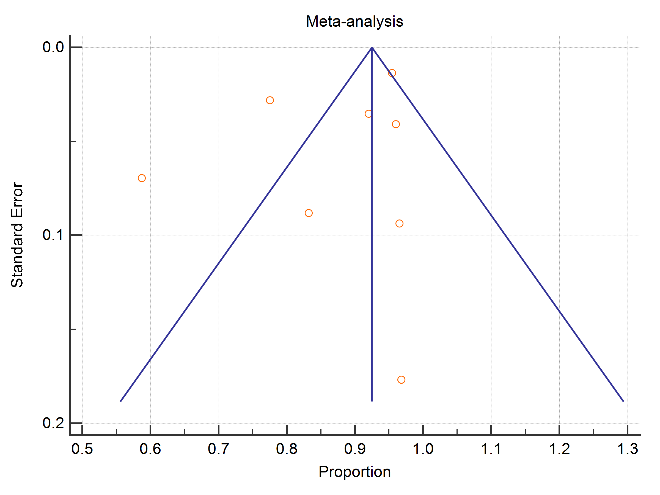 | 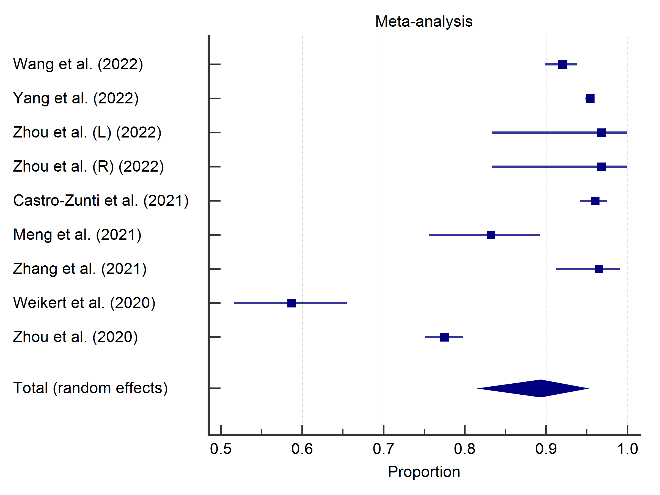 |
| Clinicians old fractures | 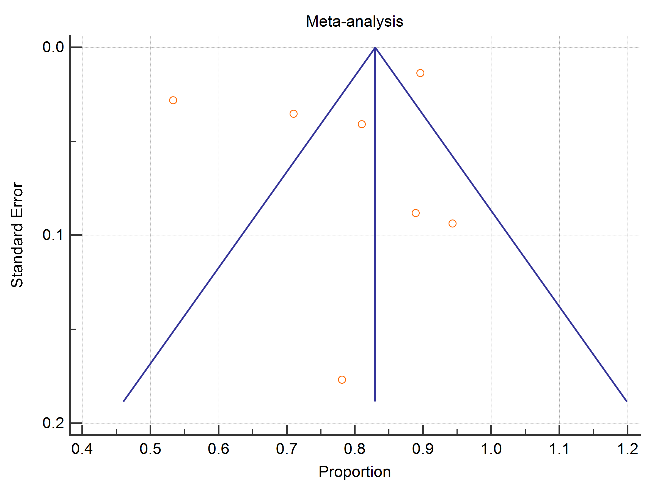 | 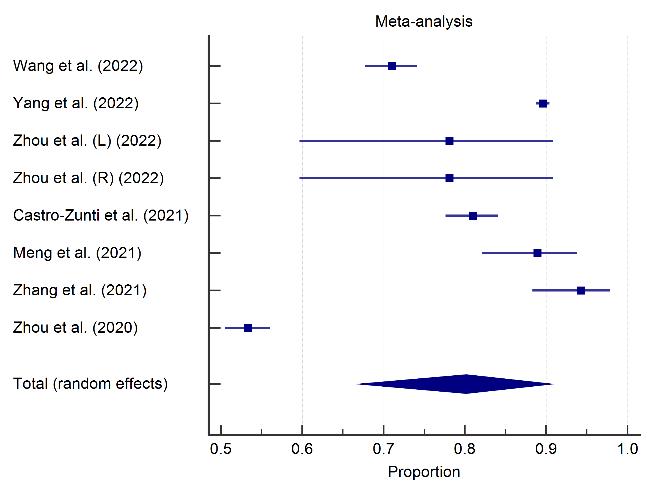 |

**SDC 8: Forest and Funnel plots of the pooled sensitivity of the detection DL models with external validation**

|  | **Funnel plot** | **Forest plot** |
| --- | --- | --- |
| **Detection model** | 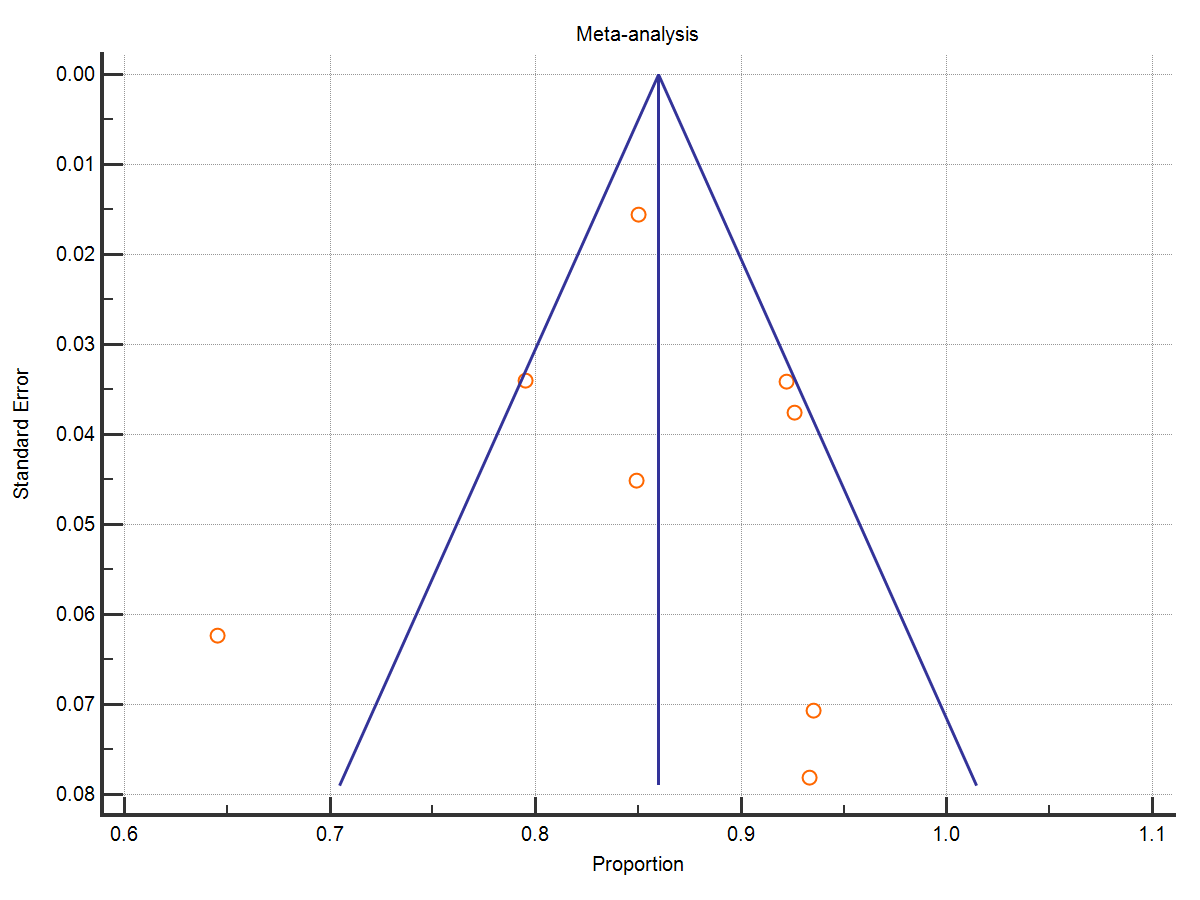 | 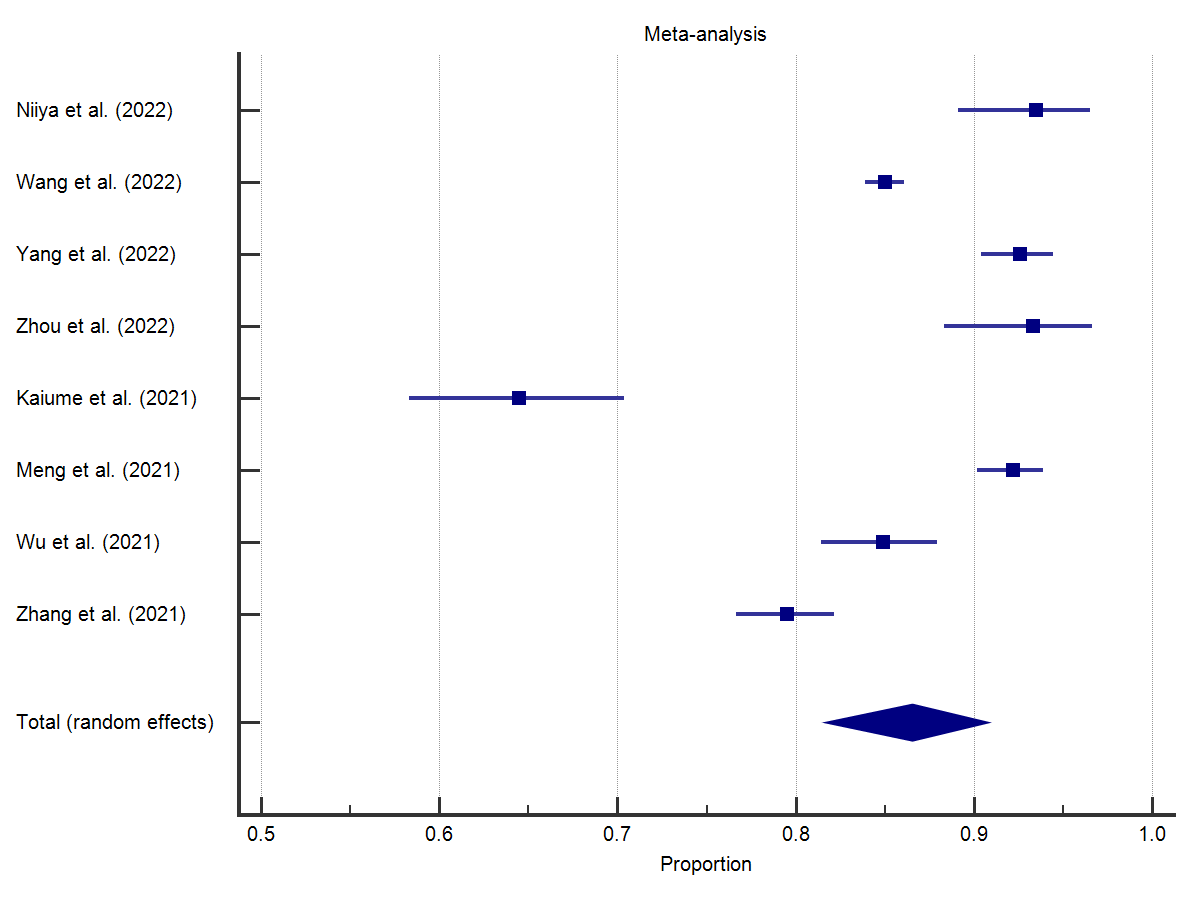 |
| **Clinicians detection model** | 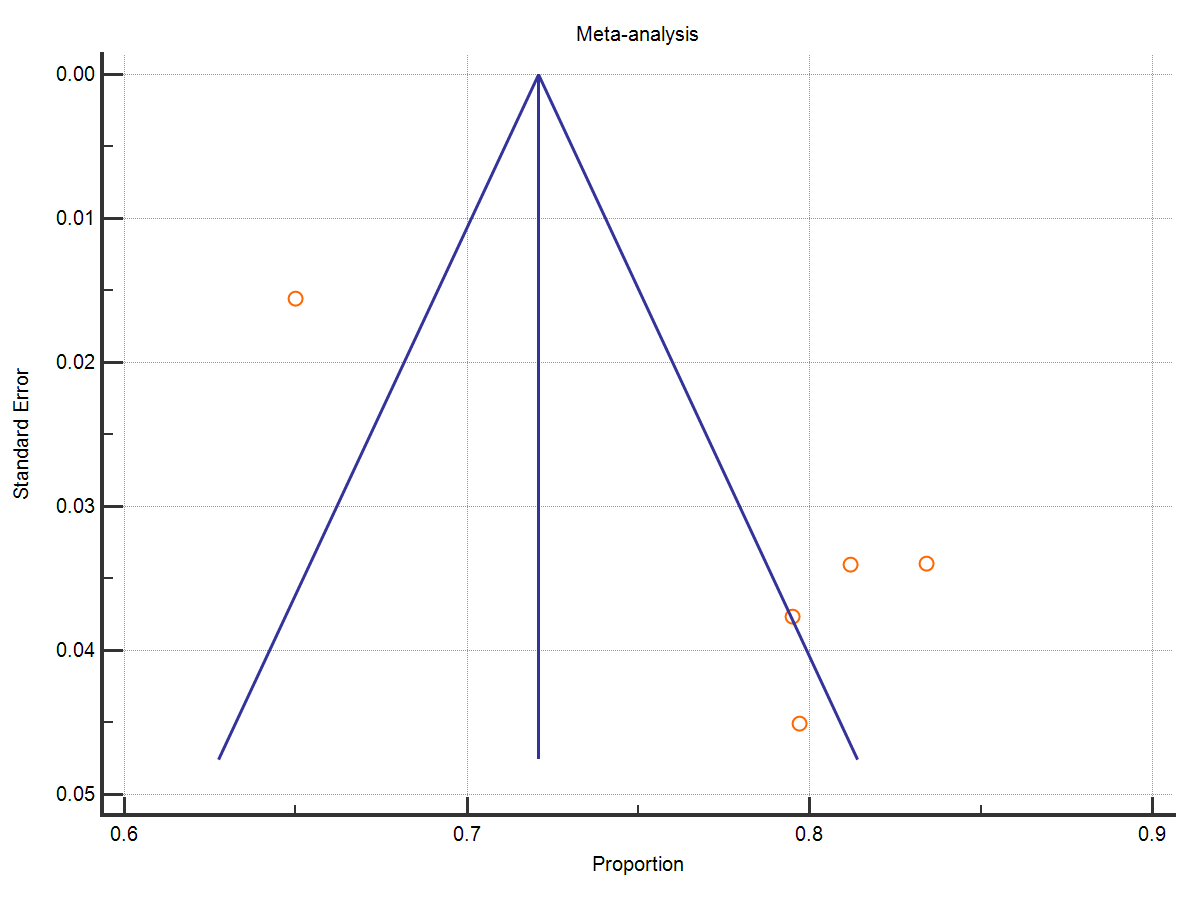 | 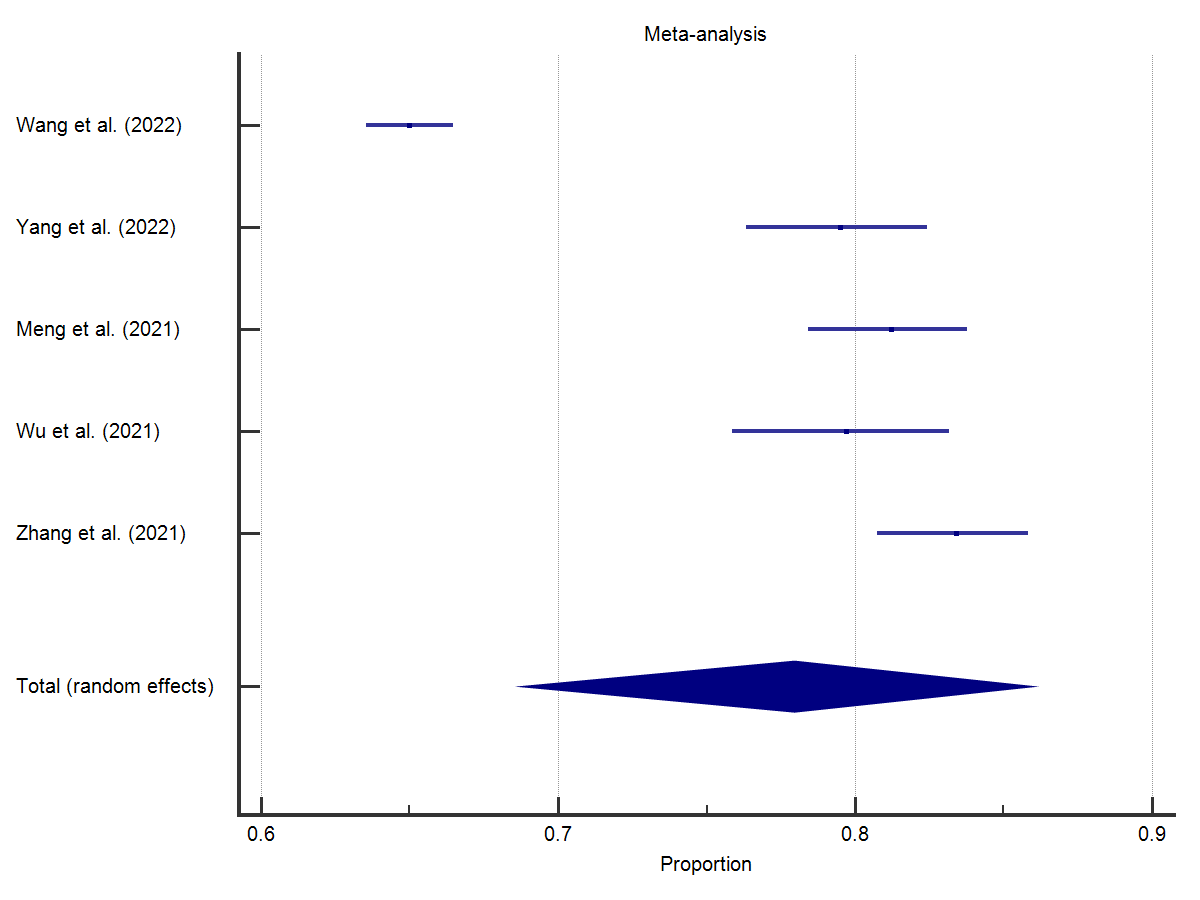 |
